# Synthesize of Bi_2_O_3_/Gln-TSC nanoparticles and evaluation of their toxicity on prostate cancer cells and expression of *CASP8*, *BAX*, and *Bcl-2* genes

**DOI:** 10.1038/s41598-022-25360-6

**Published:** 2022-12-08

**Authors:** Asal Moradi, Mohammadreza Abdihaji, Sara Barari Kouchaksaraie, Tabarek Abdulrazaq Alkinani, Aida Mahmoudi, Arash Davoudi, William Dashtmiani, Somayeh Mikaeili Ghezeljeh, Shahrzad Aghajani, Reza Ghasemian, Somayeh Maghsoomi Taramsari, Amitis Majlesi, Zahra Mahdavi Niyaki, Ali Salehzadeh

**Affiliations:** 1 Department of Biology, Rasht Branch, Islamic Azad University, Rasht, Iran; 2 Department of Biology, The Center for Genomics and Bioinformatics, Indiana University, Bloomington, IN USA; 3 Department of Biology, Science and Research Branch, Islamic Azad University, Tehran, Iran; 4 Department of Biology, Damghan Branch, Islamic Azad University, Damghan, Iran; 5 Division of Cytogenetic, Dr. Keshavarz Medical Genetics Lab, Rasht, Iran; 6 Cancer Research Laboratories, Department of Surgery, St. George Hospital, University of New South Wales, Sydney, NSW 2217 Australia; 7 Department of Medical Sciences, Faculty of Medicine, Tehran Medical Sciences, Islamic Azad University, Tehran, Iran; 8 Max Perutz Labs, Vienna Biocenter (VBC), Dr. Bohr-Gasse 9, University of Vienna, Vienna, Austria

**Keywords:** Medical research, Nanoscience and technology

## Abstract

Due to the high prevalence and considerable increase of prostate cancer, finding novel therapeutic compounds for the treatment of prostatic cancer has been the goal of many researches. In this study, we aimed to fabricate the Bismuth oxide (Bi_2_O_3_) NPs, functionalized with glutamine (Gln) and conjugated with Thiosemicarbazide (TSC). Then, the anticancer mechanism of the synthesized NPs was investigated using the cellular and molecular tests including MTT assay, Flow cytometry, Caspase-3 activity assay, Hoechst staining and Real Time PCR. The FT-IR and XRD assays confirmed the identity of the synthesized Bi_2_O_3_/Gln-TSC NPs. The size range of the synthesized spherical particles was 10–60 nm and the zeta potential was − 23.8 mV. The purity of the NPs was confirmed by EDX-mapping analysis. The Bi_2_O_3_/Gln-TSC was considerably more toxic for prostate cancer cells than normal human cells and the IC_50_ was calculated 35.4 and 305 µg/mL, respectively. The exposure to the NPs significantly increased the frequency of apoptotic cells from 4.7 to 75.3%. Moreover, the expression of the *CASP8*, *BAX*, and *Bcl-2* genes after exposure to the NPs increased by 2.8, 2.3, and 1.39 folds. Treating the cancer cells with Bi_2_O_3_/Gln-TSC increased the activity of the Caspase-3 protein and apoptotic morphological features were observed by Hoechst staining in the treated cells. This work showed that Bi_2_O_3_/Gln-TSC has considerable cytotoxicity for prostate cancer cells and could inducing both intrinsic and extrinsic pathways of apoptosis.

## Introduction

With an estimated 1,414,000 new cases, prostate cancer is considered the third most commonly diagnosed cancer, worldwide, after breast and lung cancers. This disease is involved with almost 375,000 deaths and is considered the 8th cause of cancer-associated death^1^. The current therapeutic measures include chemotherapy, radiation, and surgery. Due to the lack of specificity, low efficacy, and considerable side-effects of conventional therapeutic methods, there has been an increasing interest in the application of nanotechnology for prostate cancer diagnosis and treatment^2^. The large surface/volume ratio and morphology of the nanoparticles play a crucial role in their distribution in the body and their cytotoxic effect on cancer cells^2^. Several nanostructures have been introduced for cancer treatment; however, controversial results have been observed^3^. The low efficacy and toxicity for normal cells are considered the most limiting factors in the development of many nanoparticles (NPs) in cancer treatment. Therefore, the preparation of novel nanostructures containing several effective molecules could be a promising method for the introduction of novel and safer anticancer agents for cancer treatment.

Bismuth NPs have gained considerable attention in the biomedicine and the cosmetic industry^4^. The bactericidal, fungicidal, antiparasitic and anti-biofilm properties of Bismuth NPs coated with lipophilic dimercaptopropanol NPs have been extensively studied in several papers^5,6^. The in vitro cytotoxicity of Bismuth NPs coated with cellulose nanofibers was evaluated by MTT assay on mouse breast cancer cell lines^7^. Recently, Hamood et al.^8^ evaluated the anticancer activity of Bi@PVP NPs on the human MCF-7 breast cancer cell line. The cytotoxic effects of the BisBAL NPs were specifically studied on erythrocytes^9^, epithelial cells^10^, fibroblasts and cancer cells (cervical, prostate, colon in humans)^11^. The generation of intracellular reactive oxygen species (ROS) and activation of cellular pro-apoptotic pathways have been the major cytotoxic features of these NPs that could be employed for cancer chemotherapy^4–12^. However, the accumulation in the body and also, cytotoxic side effects are considered the major drawback of them to be used in medical applications.

Conjugation of therapeutic molecules is a novel approach that could be used to increase the efficacy of NPs in cancer treatment and reduce their cytotoxic side effects. Glutamine plays a critical role in cancer metabolism. An excessive uptake and consumption of glutamine by a variety of cancer cells have been reported^13,14^. Therefore, conjugation of nanoparticles to glutamine may increase the efficacy of the complex molecule against cancer cells. Also, glutamine could be used for the functionalization of NPs and their conjugation to therapeutic molecules to increase their anticancer activity^15,16^.

Thiosemicarbazides (TSCs) and their derivatives have shown considerable therapeutic potential as antiviral, antibacterial, and anticancer agents^13^. TSCs could interrupt the replication of cellular nucleic acids and inhibit cell proliferation^14^. Therefore, the conjugation of TSCs to NPs may increase the activity of the complex compared with either agent. In this regard, the present work aimed to fabricate bismuth oxide nanoparticle functionalized with glutamine and conjugated with TSC and to characterize its effect on prostate cancer cells and expression of *CASP8*, *BAX,* and *Bcl-2* genes.

## Materials and methods

### Synthesis of Bi_2_O_3_ and Bi_2_O_3_/Gln-TSC NPs

**Scheme 1:**
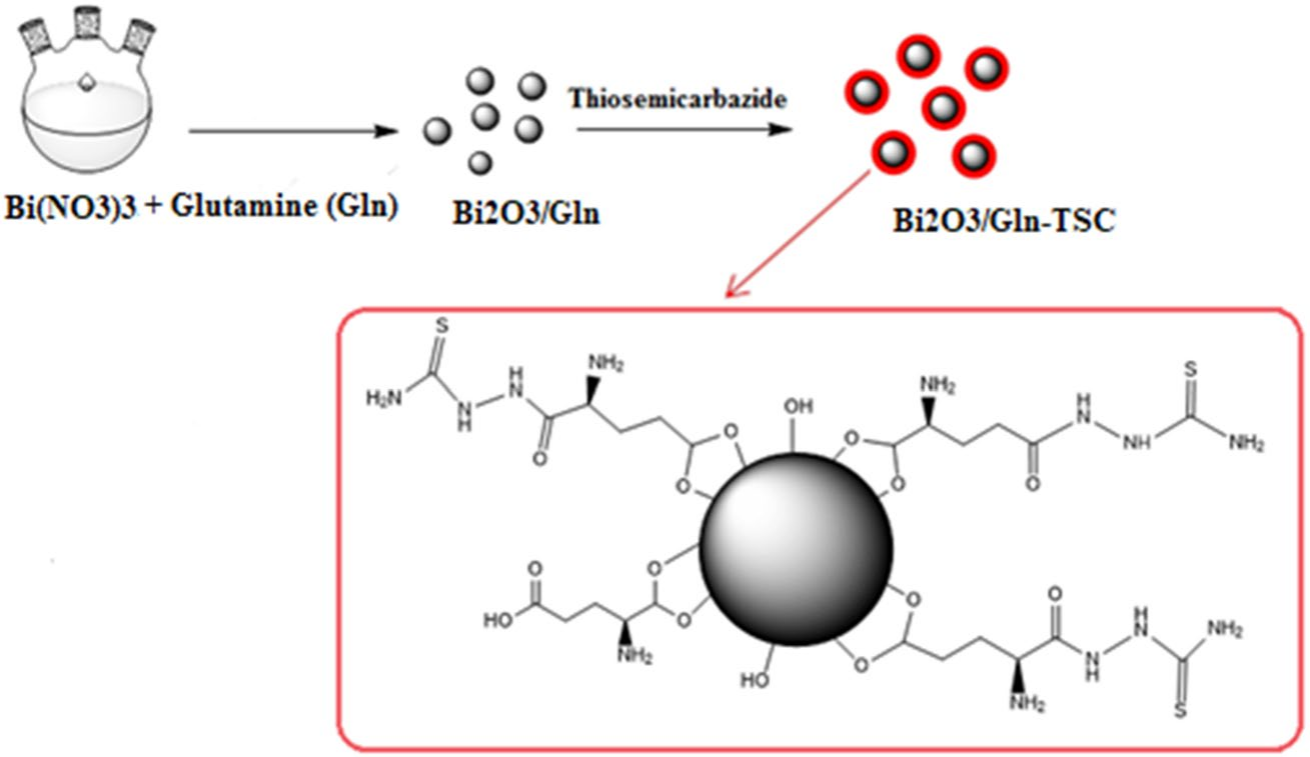
Schematic diagram illustrating the synthetic procedure of Bi_2_O_3_/Gln-TSC NPs.

To prepare Bi_2_O_3_/Gln NP, at first, 300 mg of Bismuth (III) nitrate and 150 mg of Glutamine (Sigma-Aldrich) were dissolved in 150 mL of dissolved water and the pH was adjusted to 11.0 using 10% NaOH solution. Next, the mixture was heated at 80 °C for 2 h and the resulting dark pellet was harvested, washed, and dried at 70 °C for 8 h (Scheme 1).

For the synthesis of Bi_2_O_3_/Gln-TSC, 500 mg of the synthesized Bi_2_O_3_/Gln and 200 mg of TSC were suspended in 150 mL 96% ethanol and sonicated for 45 min at 40 °C. The mixture was maintained at 40 °C for 24 h with continuous shaking. The prepared Bi_2_O_3_/Gln-TSC NPs were harvested by centrifugation and dried at 70 °C for 8 h^17^.

Physicochemical assays were performed to characterize the prepared Bi_2_O_3_/Gln-TSC. FT-IR analysis was performed using a Nicolet IR 100 FT-IR spectrophotometer in a range of 500–4000 cm^−1^. Also, the X-ray diffraction (XRD) pattern of the NP was determined to assess the crystalline structure of the particles (Co-Ka X-radiation, k = 1.79 Å). Scanning (Zeiss-Sigma VP, Germany) and transmission electron microscopy (Zeiss-EM10C, Germany) were used to determine the size range and morphology of the NP. The purity of the Bi_2_O_3_/Gln-TSC was assessed using the EDX-mapping analysis. Further, Zeta potential and DLS analyses (Malvern Instruments Ltd, Ver. 6.32) were used to assess the stability and aggregation degree of the NP.

### MTT assay

The PC3 prostate cancer cell line and HEK293 cell line (normal human cell line) were obtained from the National Cell Bank of Pasteur Institute of Iran. The cytotoxic effect of Bi_2_O_3_/Gln-TSC on PC3 prostate cancer cells and HEK293 cells was evaluated by 2-(4,5-dimethythiazol-2-yl) -2,5-diphenyltetrazolium bromide (MTT) assay^18^. For culture maintenance, the cells were cultured on standard Dulbecco’s modified Eagle’s medium (DMEM) with 10% fetal bovine serum (FBS), 100 μg/mL streptomycin, and 100 units/mL penicillin solution at 37 °C in the presence of 5% humidified CO_2_ in air. Approximately, 1 × 10^4^ cells were plated in each well of a 96- well plate and cultured for 24 h. Thereafter, the PC3 and HEK293 cells were exposed to a series of 15.6–500 µg/mL concentrations of Bi_2_O_3_/Gln-TSC at 37 °C in the presence of 5% humidified CO_2_ for 24 h. The untreated cancer cells were assayed as control. After completion of incubation time, the untreated and Bi_2_O_3_/Gln-TSC treated cells were incubated by 100 μL MTT solution (0.5 mg/ mL) at the same condition for 4 h. In each well, 100 μL of dimethyl sulfoxide (DMSO) was added to dissolve the formazan crystal formed in the reaction. The plate was shaken for 30 min and then, the optical absorbance at 590 nm was measured by a microplate ELISA reader instrument (Bio-Rad, Hercules, CA, USA). The ratios of OD value of treated Bi_2_O_3_/Gln-TSC samples to the untreated samples were used to calculate the percentage of cell viability. The following formula was used to calculate the 50% inhibitory concentration of the NP^19^:

1 $$ {\text{Growth}}\;{\text{inhibition}}\% = 100 - \left[ {\frac{{{\text{Absorbance}}\;{\text{of}}\;{\text{sample}}}}{{{\text{Absorbance}}\;{\text{of}}\;{\text{control}}}} \times 100} \right] $$

### Flow cytometry analysis

The PC3 cells were treated with Bi_2_O_3_/Gln-TSC NPs at IC_50_ concentration. After 24 h incubation at 37 °C, the cells were washed and treated with propidium iodide and Annexin V (Roche, Germany). Then, flow cytometry analysis was performed (Partec flow cytometry device, Germany) to determine the frequency of apoptotic cells in NPs treated and control cells.

### Quantitative PCR (qPCR)

The effect of Bi_2_O_3_/Gln-TSC on the expression of *Bcl-2*, *BAX*, and *CASP8* genes in prostate cancer cells was investigated by SYBR green quantitative PCR assay. The cancer cells (about 5000 cells) were treated with the NPs at IC_50_ concentration for 24 h, total RNA of the cells was extracted using the TriZol reagent (Sigma-Aldrich), and cDNA was synthesized by SinaClone cDNA synthesis kit (Iran). The *GAPDH* gene was used as an internal reference gene. The sequence of the primers used in this study was presented in Table 1. Data analysis was performed according to the 2^−ΔΔCT^ method^20^.

**Table 1:** Sequence of the primers used in this study (The efficiency of primers was provided in supplementary file).

Primer	Sequence (5′–3′)	Product size (bp)	References
*BAX-forward*	TTGCTTCAGGGTTTCATCCA	113	^34^
*BAX-reverse*	AGACACTCGCTCAGCTTCTTG		
*CASP8-forward*	GACTGGATTTGCTGATTACCTACCTAA	143	^37^
*CASP8-reverse*	CCTCAATTCTGATCTGCTCACTTCT		
*Bcl-2-forward*	TGGCCAGGGTCAGAGTTAAA	147	^34^
*Bcl-2-reverse*	TGGCCTCTCTTGCGGAGTA		
*GAPDH-forward*	CCCACTCCTCCACCTTTGAC	75	^37^
*GAPDH-reverse*	CATACCAGGAAATGAGCTTGACAA		

### Activity of Caspase-3

The activity of Caspase-3 protein in Bi_2_O_3_/Gln-TSC treated and control cells was measured using the method described by Salehzadeh et al.^21^. After exposure of PC3 cells with IC_50_ concentration of NP, the cells were lysed and the cell supernatant was treated with DEVD-pNA (CASP3C, Sigma-Aldrich). Finally, the optical density was measured at 405 nm.

### Hoechst staining

To elucidate the nuclear damages, prostate cancer cells were treated with IC_50_ concentration of Bi_2_O_3_/Gln-TSC NPs for 24 h. Then, the NPs treated and control cells were subjected to the Hoechst 33,258 solution for 5 min and examined under a fluorescent microscope^18^.

### Statistical analyses

Significant differences between the NPs treated and control cells were assessed using the SPSS 16.0 software. One-way analysis of variance (ANOVA) along with Post hoc analysis according to Tukey's Test method was used to uncover specific differences between group means, when ANOVA test was significant. The assays were performed in triplicates and *p*-values less than 0.05 were considered statistically significant.

## Results

### Physicochemical features of NP

**Figure 1:**
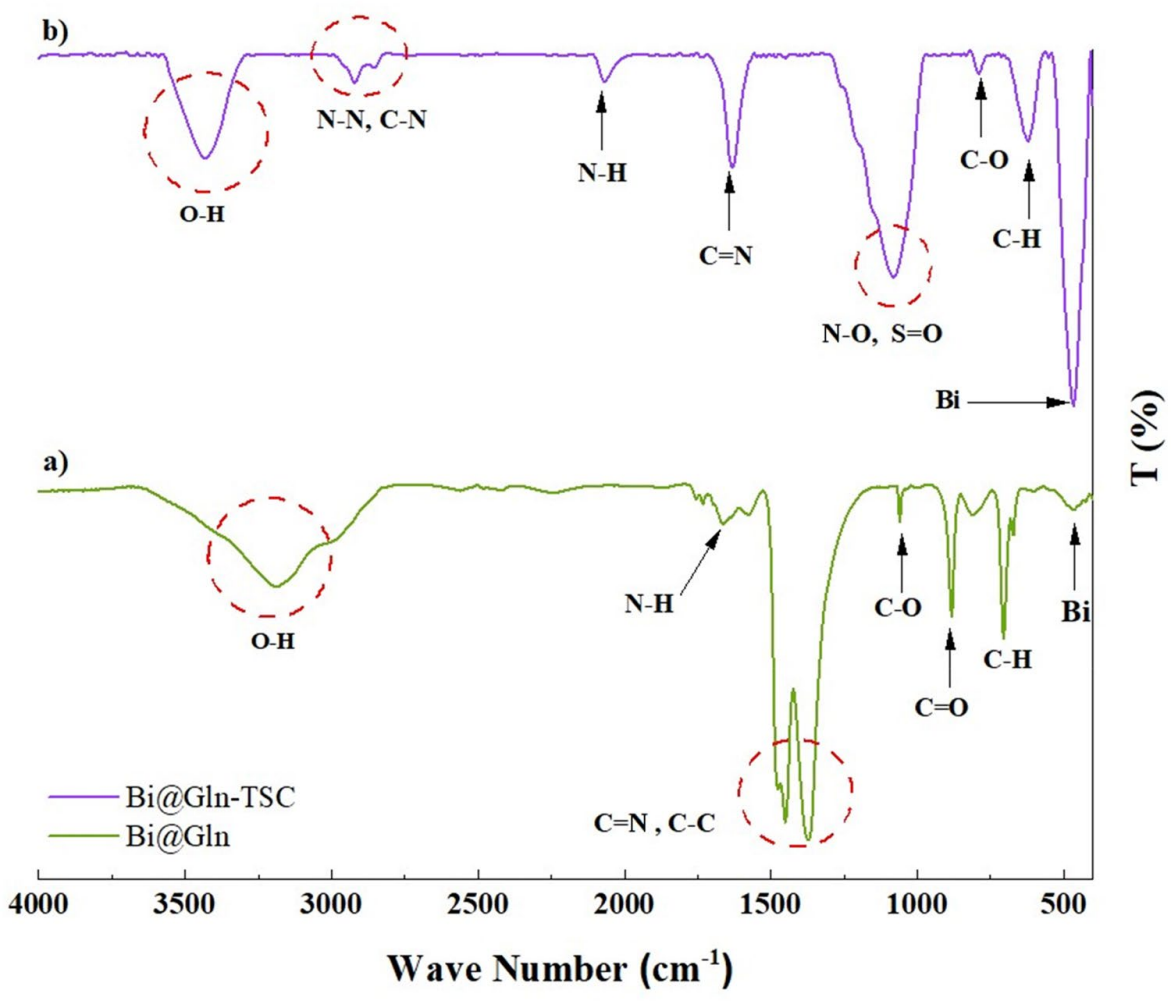
FT-IR analysis of (**a**) Bi_2_O_3_/Gln and (**b**) Bi_2_O_3_/Gln-TSC NPs.

FT-IR analysis was performed to evaluate the functional groups of Bi_2_O_3_/Gln and Bi_2_O_3_/Gln-TSC NPs. The FT-IR spectra of Bi_2_O_3_/Gln revealed two peaks at 482 and 745 cm^−1^ that are associated with the Bismuth atom. Also, the peaks observed at 1124, 1283, and 1383 cm^−1^ contribute to the C-N, C-O, and CH_3_ bonds. The peaks at 1635, 1729, and 3634 cm^−1^ are associated with the N–H, O=C, and stretching O–H bonds, respectively^22^. Assessment of the FT-IR peaks of Bi_2_O_3_/Gln-TSC NPs indicated two peaks at 494 and 604 cm^−1^ associated with the Bismuth atom, and three peaks at 743, 745, and 974 cm^−1^ that are associated with N–H, C–H, and N–H bonds, respectively. Also, the peaks at 1073, 1125, 1340, and 1384 cm^−1^ contribute to the C=S, C–N, C–O, and stretching N–O bonds, respectively. Moreover, the peaks at 1611, 1536, 1729, and 2096 cm^−1^ are associated with the N=O, C–C, C=N, and R–N=C=S bonds, respectively. The peak in the range of 3000–4000 cm^−1^ are associated with the O–H bond (Fig. 1).

**Figure 2:**
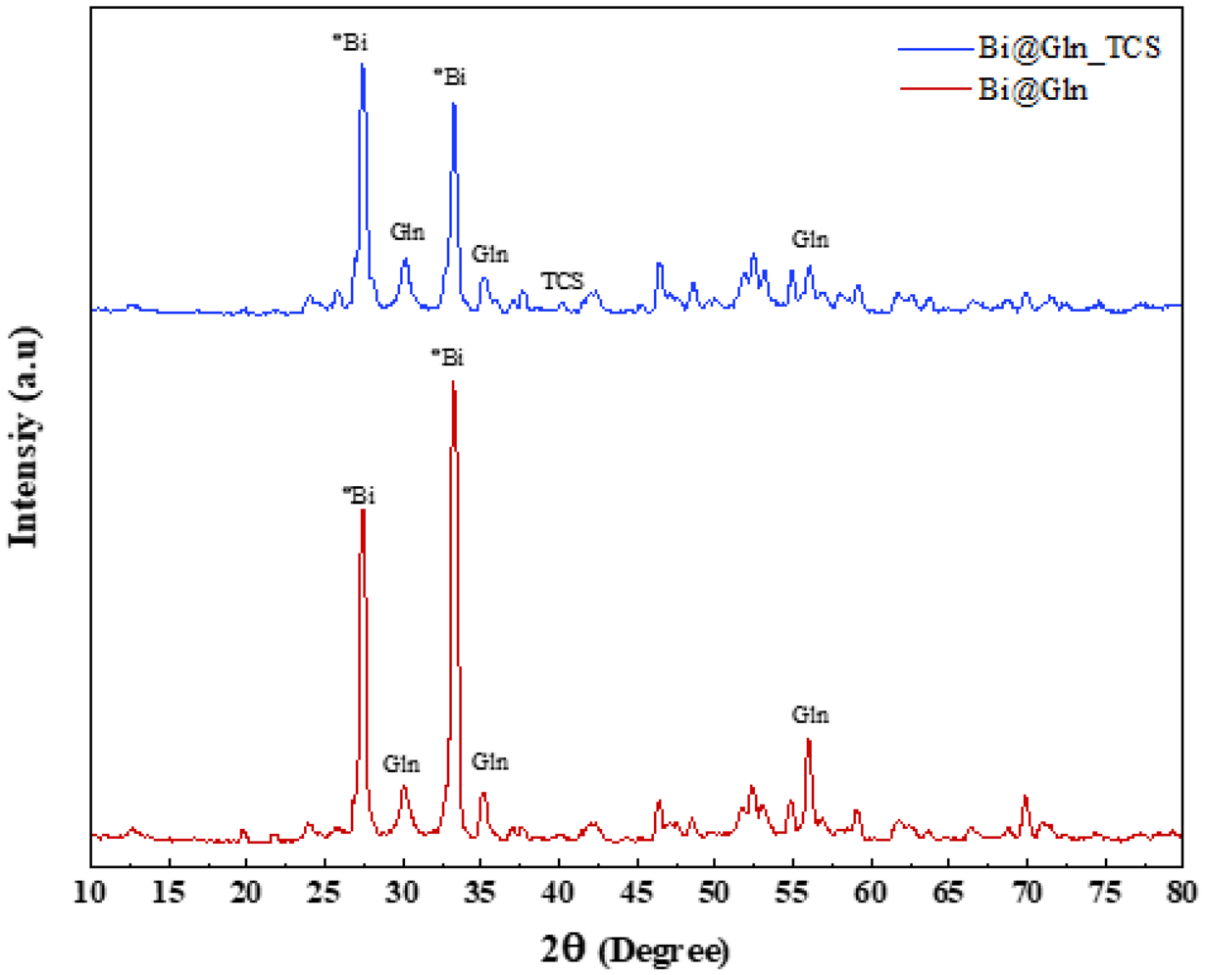
XRD pattern of the synthesized NPs.

Considering the XRD pattern of Bi_2_O_3_/Gln-TSC, the peaks at 2θ of 27.3 and 34° are associated with the Bismuth NP and comply with the JCPDS No. 01-058-1330^23^. Also, the peaks that appeared at 2θ of 32, 35, and 55.2 contribute to the Glutamine molecule of the fabricated NPs^24^. Considering the amorphous nature of TSC, two broad peaks at 2θ of 40 and 72, and also, the overall reduction of the peak intensity of Bi_2_O_3_/Gln-TSC NPs (compared with the Bi_2_O_3_/Gln), the presence of TSC molecules could be concluded^25^ (Fig. 2).

**Figure 3:**
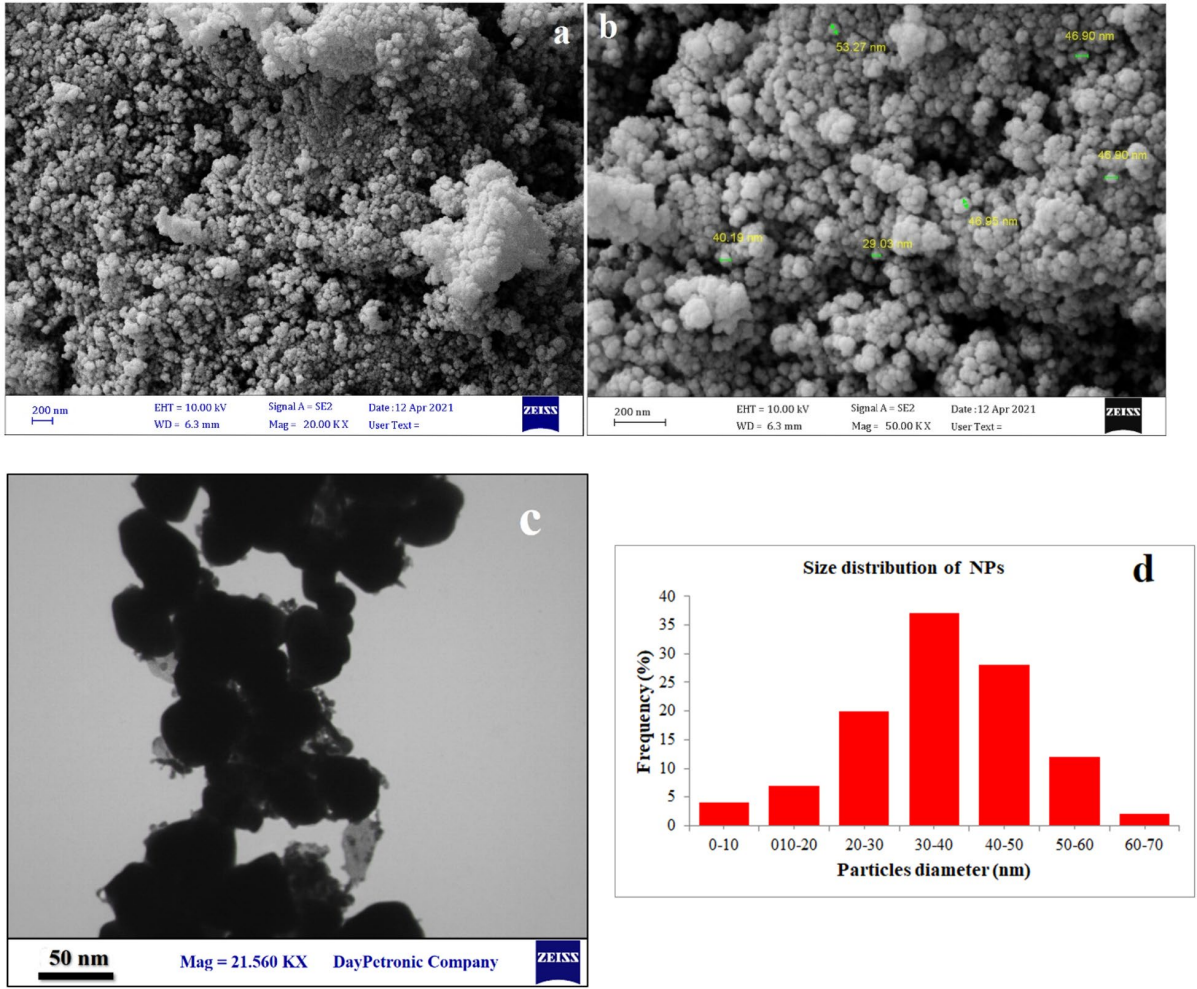
SEM (**a**,**b**), and TEM (**c**) images of Bi_2_O_3_/Gln-TSC NPs. (**d**) Particle size distribution of synthesized NPs.

**Figure 4:**
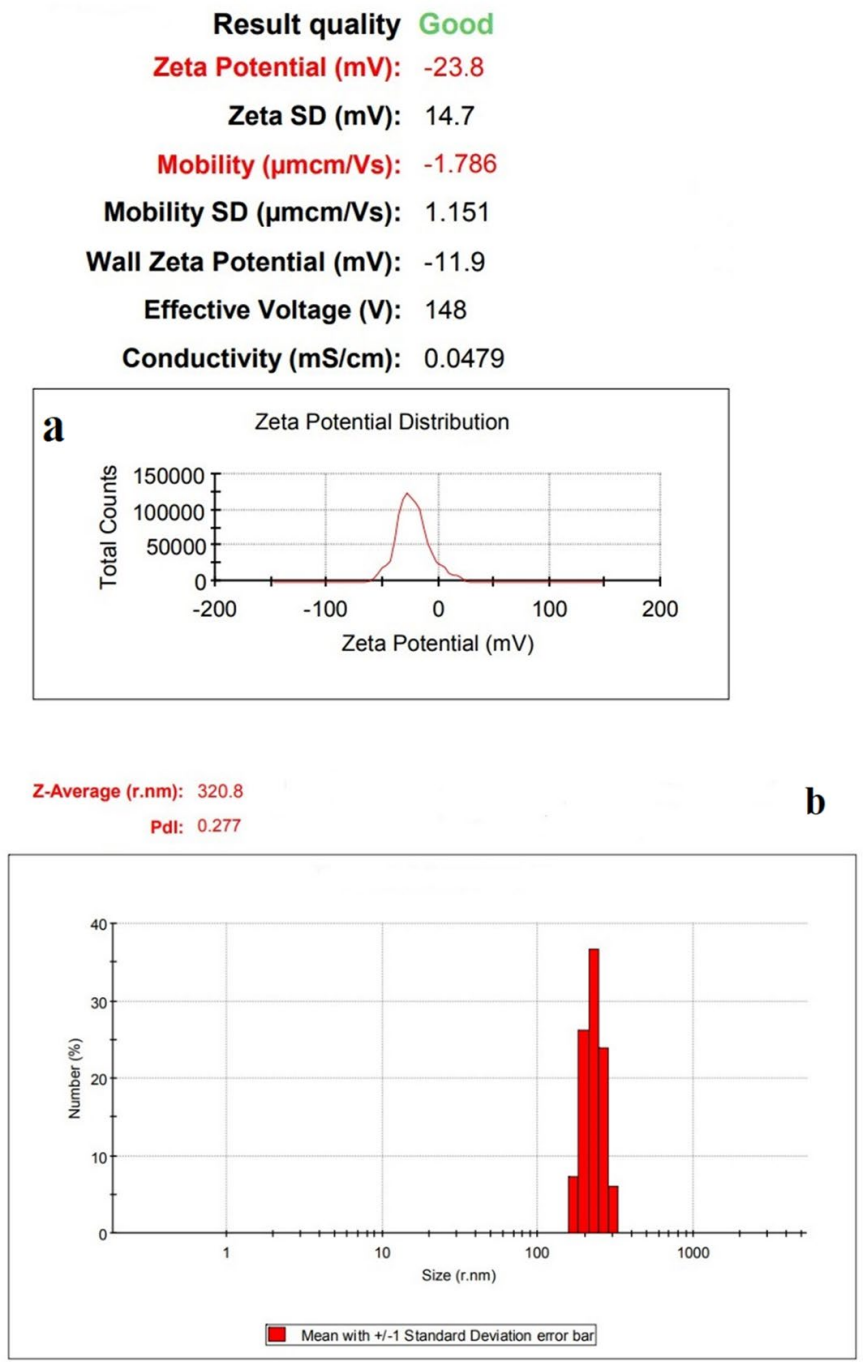
Zeta potential (**a**) and DLS (**b**) analyses of Bi_2_O_3_/Gln-TSC NPs.

**Figure 5:**
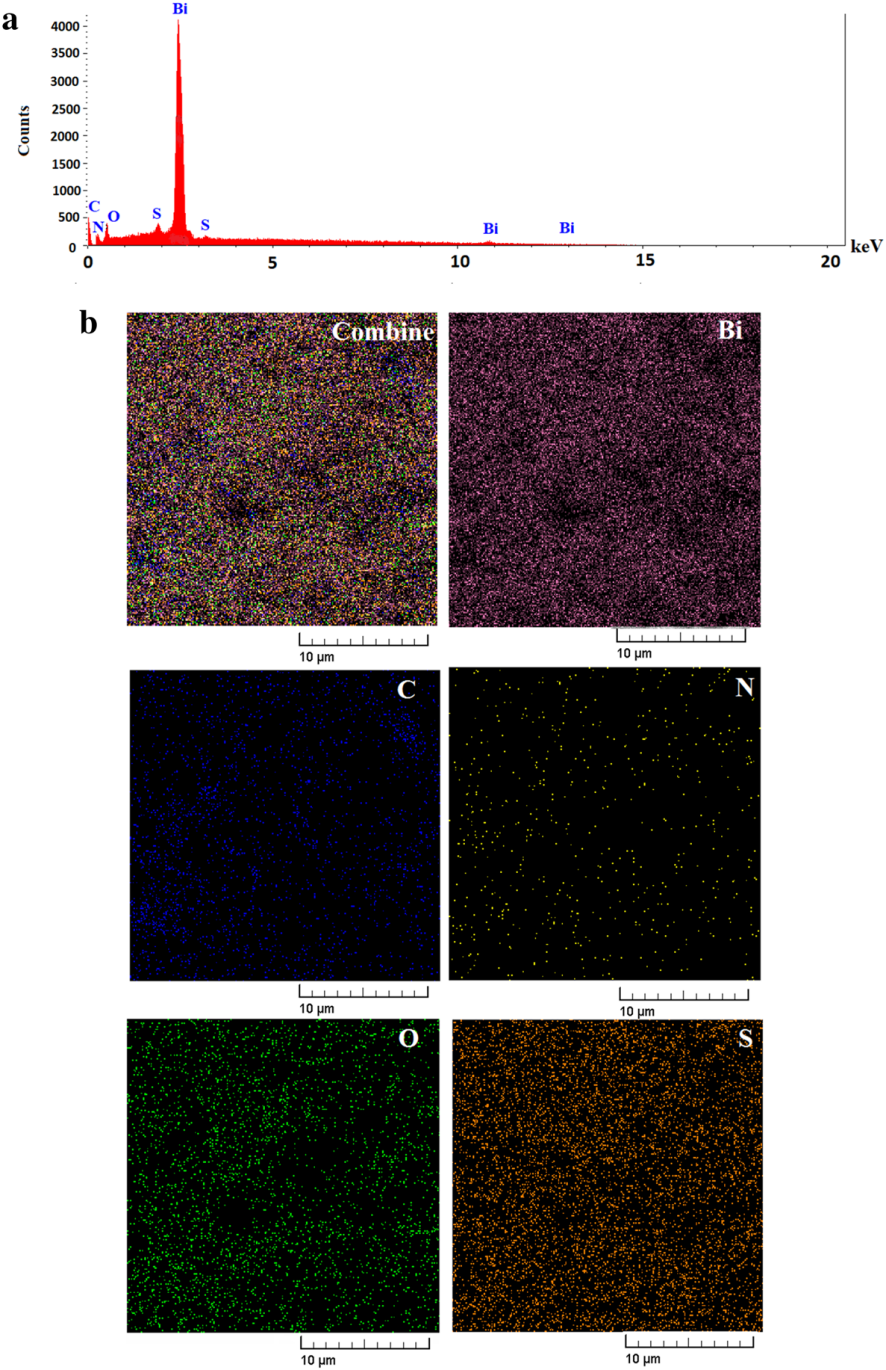
EDS-mapping of Bi_2_O_3_/Gln-TSC NPs. The spectrum shows the presence of Bi, O, N, C and S elements in the final product (Bi_2_O_3_/Gln-TSC) (**a**). Also, the maps blow the spectrum show the distribution of Bi, O, N, C and S elements in the final product (**b**).

The SEM and TEM images of Bi_2_O_3_/Gln-TSC NPs revealed that the synthesized NPs were almost spherical, with low aggregation, and with a size range of 10–60 nm. Figure 3 presents the electron microscopy images of the fabricated NPs. Moreover, the Zeta potential and hydrodynamic size of Bi_2_O_3_/Gln-TSC were measured − 23.8 mV (Zeta SD = 14.7 mV) and 320 nm (PDI = 0.227), respectively that indicate the proper stability and low aggregation of the NPs. The results were displayed in Fig. 4. The EDX-mapping analysis revealed that the NPs contained Bi, C, N, O, and S atoms and were free of impurities (Fig. 5 and Table 2).

**Table 2:** Elemental composition results of Bi_2_O_3_/Gln-TSC NP in EDS-mapping analysis.

Elements	Line	Int	Error	K	Kr	W%	A%	ZAF	Pk/Bg
C	Ka	65.7	151.1037	0.0211	0.0150	4.98	9.63	0.3005	74.93
N	Ka	33.5	151.1037	0.0108	0.0077	1.80	2.98	0.4283	29.67
O	Ka	1219.1	151.1037	0.3978	0.2824	46.28	67.20	0.6101	987.79
S	Ka	70.5	74.3259	0.0257	0.0182	2.14	1.55	0.8504	5.23
Bi	Ka	1207.2	0.6624	0.5446	0.3866	44.80	18.63	0.8630	44.23
				1.0000	0.7098	100.00	100.00		

### MTT assay

**Figure 6:**
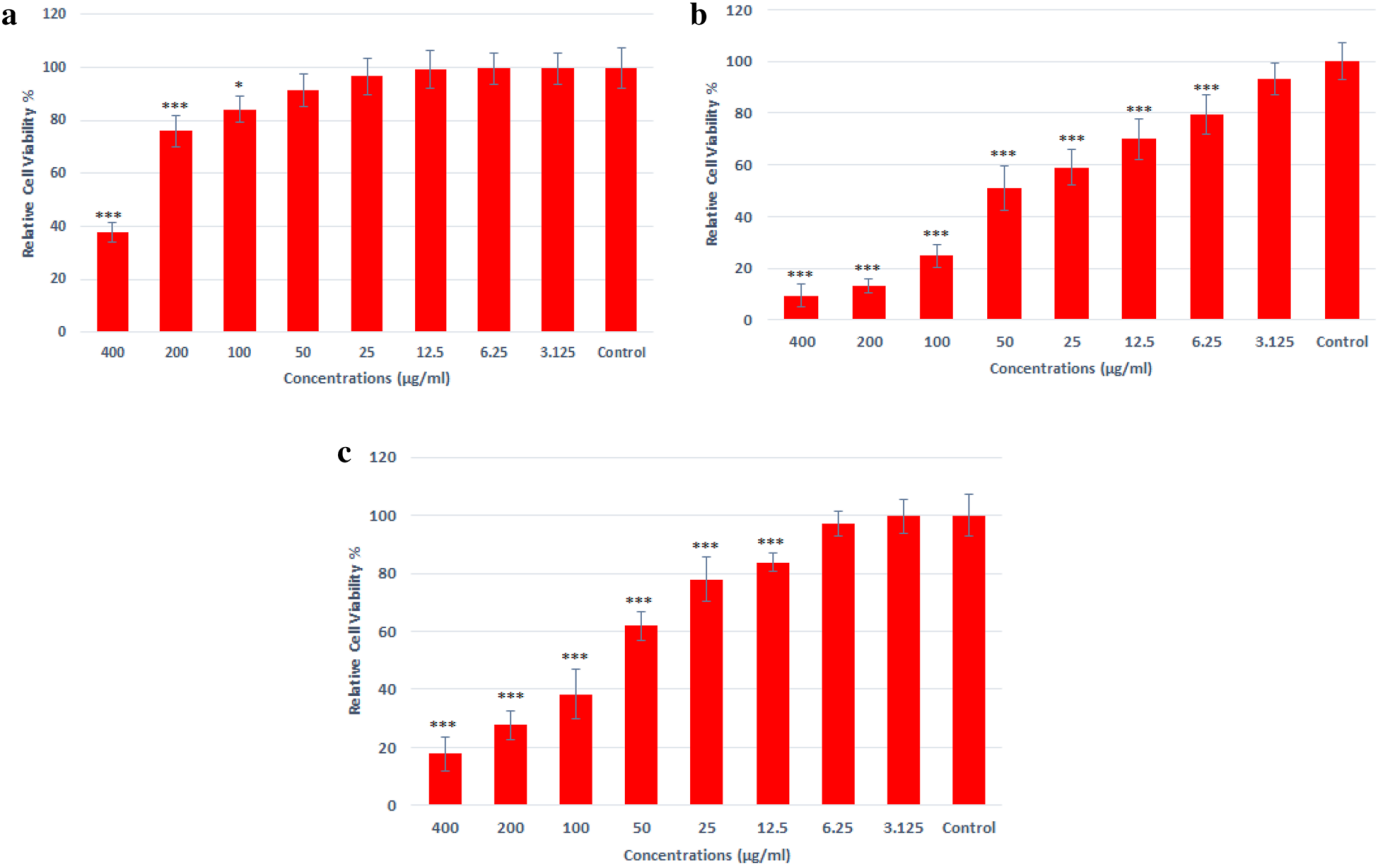
Viability of normal human cells (**a**) and prostate cancer cells (**b**) after treatment with Bi_2_O_3_/Gln-TSC NPs c: Viability of prostate cancer cells after treatment with Bi_2_O_3_/Gln NPs (Data are normalized to the untreated cells and reported as mean ± SD. Asterisks (*) indicate a significant difference with the control group (* = *P* < 0.05, ** = *P* < 0.01, *** = *P* < 0.001).

The cytotoxic effect of Bi_2_O_3_/Gln-TSC for cancer cells and normal human cells was investigated by MTT assay. According to the results, the Bi_2_O_3_/Gln-TSC NPs were more toxic for cancer cells than HEK293 cells with IC_50_ of 35.4 and 350 µg/mL, respectively. Furthermore, the IC_50_ of Bi_2_O_3_/Gln NPs on cancer cell line was 74.8 µg/mL. Therefore, the cancer cells were considerably more susceptible to the synthesized NPs than normal human cells. Our results showed that at 6.25 µg/mL and higher concentrations, Bi_2_O_3_/Gln-TSC significantly reduced the viability of cancer cells. The results were presented in Fig. 6.

### Flow cytometry analysis

**Figure 7:**
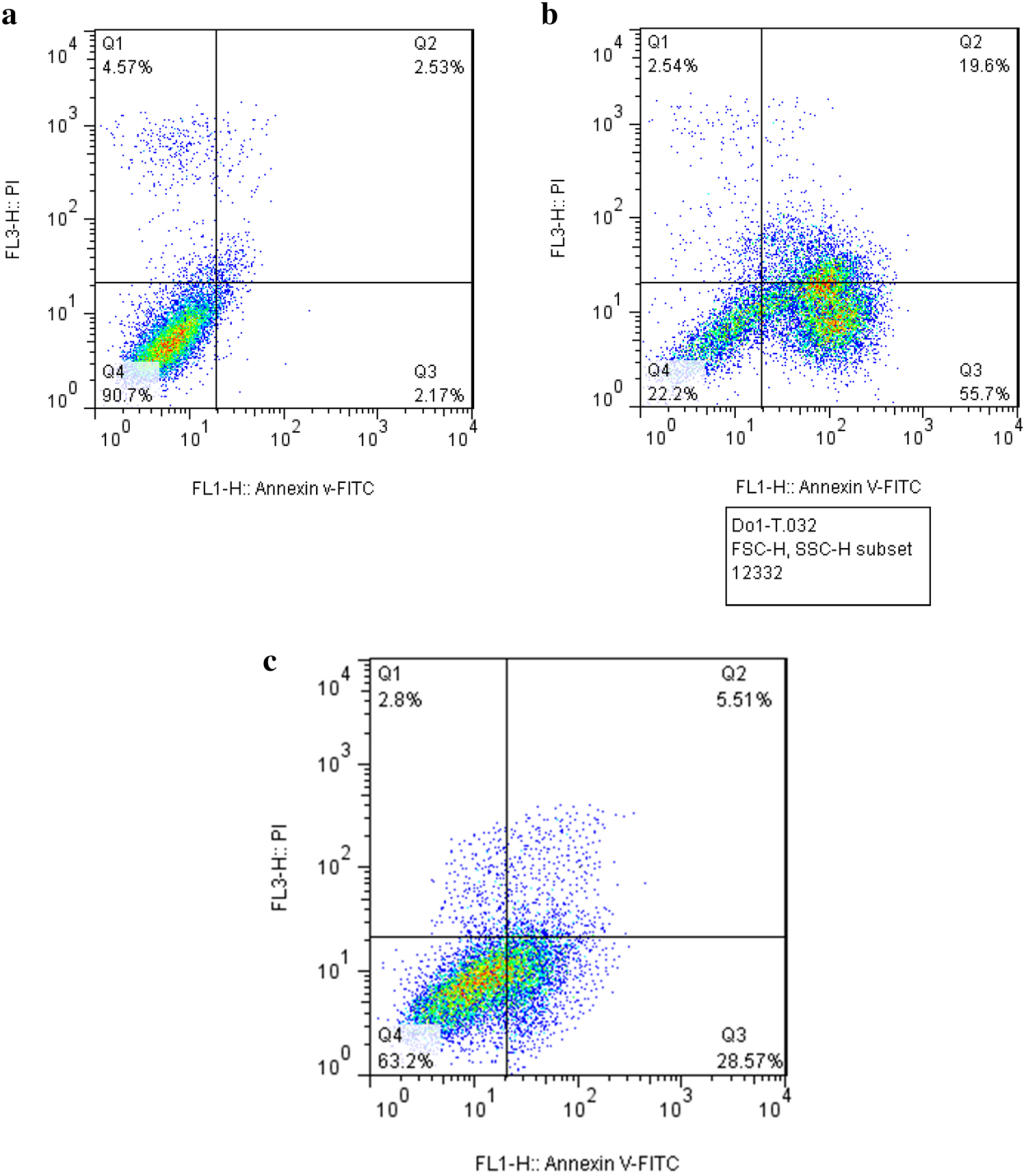
Flow cytometry of control (**a**), Bi_2_O_3_/Gln-TSC NPs treated cells (**b**) and Bi_2_O_3_/Gln NPs treated cells (**c**) (Q1: Necrotic cells, Q2: Late apoptosis, Q3: Early apoptosis, Q4: Live cells).

Flow cytometry assay was performed to investigate the effect of exposure to Bi_2_O_3_/Gln-TSC NPs on the apoptosis of prostate cancer cells. Our results showed that after exposure to the NPs, the frequency of apoptotic cells considerably increased compared with the control cells. The frequency of the cells with early and late apoptosis in control PC3 cells was measured 2.53 and 2.17%, respectively, while, after treating the cells with Bi_2_O_3_/Gln-TSC NPs the frequency of early and late apoptosis increased to 19.6 and 55.7%, respectively. Also, about 2.54% of the treated cancer cells with NPs were in necrosis condition and 22.2% of them were alive. In treating the cancer cells with Bi_2_O_3_/Gln NPs the frequency of early and late apoptosis was 28.57 and 5.51%, respectively. Furthermore, about 2.8% of the treated cancer cells with Bi_2_O_3_/Gln NPs were in necrosis condition and 63.2% of them were alive. The results were presented in Fig. 7.

### Genes expression

**Figure 8:**
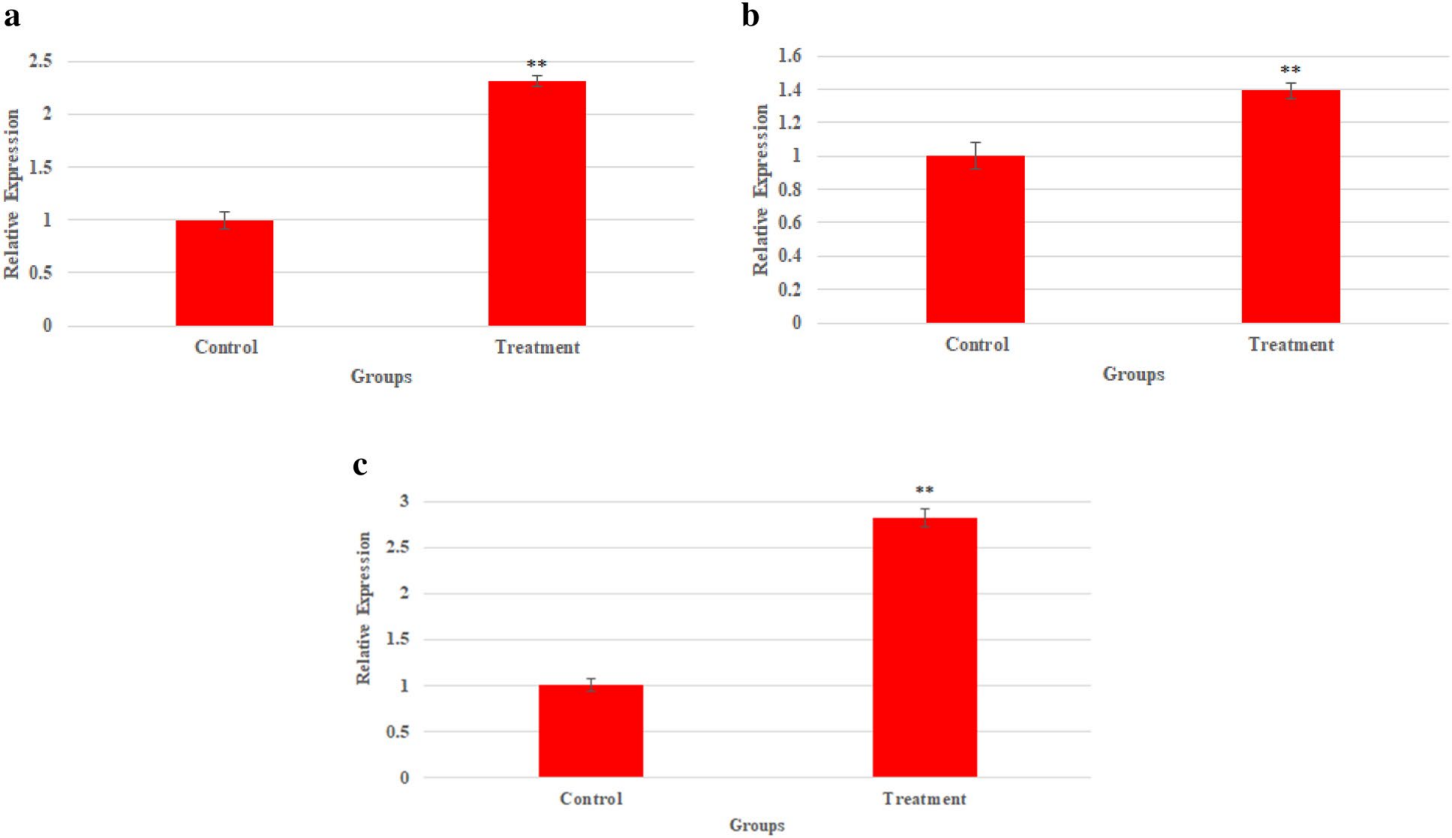
Relative expression of the (**a**) *BAX*, (**b**) *Bcl-2*, and (**c**) *CASP8* gene in Bi_2_O_3_/Gln-TSC NPs treated cells. (Data are normalized to untreated cells and reported as mean ± SD. Asterisks (*) indicate a significant difference with the control group (* = *P* < 0.05, ** = *P* < 0.01, *** = *P* < 0.001).

The expression of the apoptosis pathway genes, including *BAX, Bcl-2* and *CASP8* genes in NPs treated and control PC3 cells was investigated. The qPCR assay showed that treating the cells with the NPs considerably up-regulated the *BAX* gene, with an increase of 2.3 folds. Also, the expression of the *Bcl-2* gene was slightly increased by 1.39 folds, after exposure to Bi_2_O_3_/Gln-TSC. The ratio of *BAX to Bcl-2* expression was equal to 1.65. The mRNA level of *CASP8* gene was also considerably increased by 2.8 folds in the treated cells, compared with the control PC3 cells. The results were displayed in Fig. 8.

### Caspase-3 activity assay

**Figure 9:**
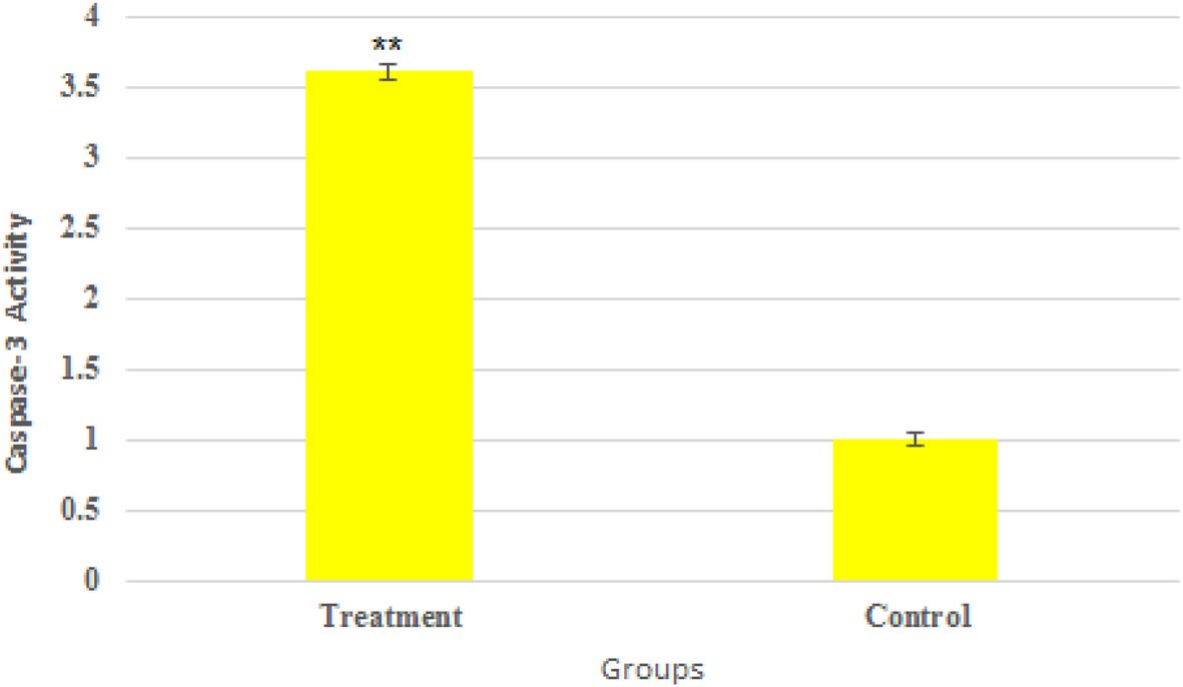
The activity of Caspase-3 enzyme in NPs treated and control cells. (Data are normalized to untreated cells and reported as mean ± SD. Asterisks (*) indicate a significant difference with the control group (* = *P* < 0.05, ** = *P* < 0.01, *** = *P* < 0.001).

The activity of caspase-3 protein in prostate cancer cells was significantly increased than in control cells. As presented in Fig. 9, we observed that the exposure to Bi_2_O_3_/Gln-TSC could significantly increase the caspase-3 activity by 3.6 folds.

### Hoechst staining

**Figure 10:**
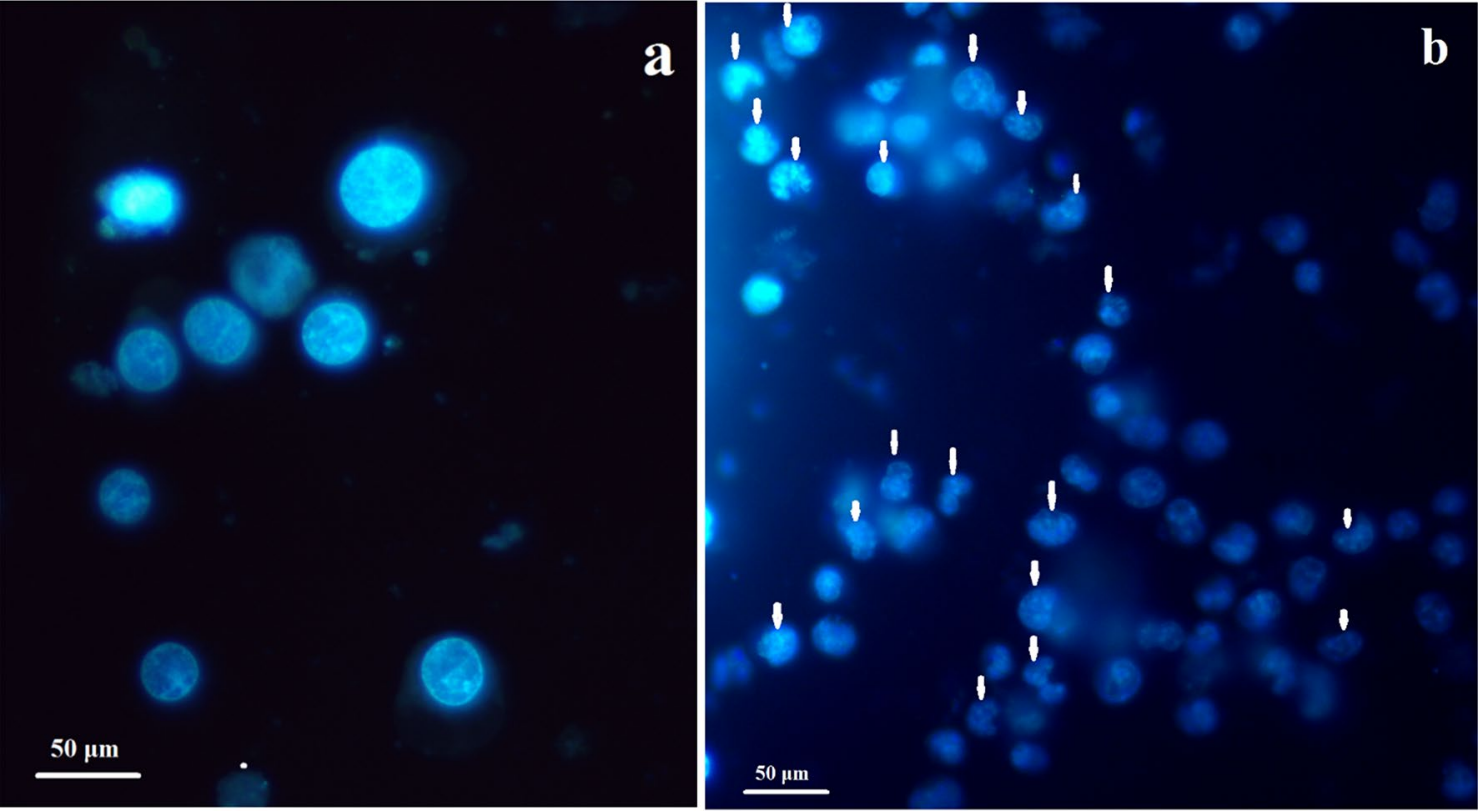
Hoechst staining of (**a**) control, and (**b**) Bi_2_O_3_/Gln-TSC NPs treated cells. The arrows show the chromatin condensation and fragmentation and also the appearance of apoptotic bodies in the cells treated with Bi_2_O_3_/Gln-TSC NPs.

Hoechst staining assay was performed on the Bi_2_O_3_/Gln-TSC treated and control cells to determine the possible nuclear damages caused form NPs exposure. The results revealed the chromatin condensation and fragmentation and also the appearance of apoptotic bodies as the major outcomes of exposure to Bi_2_O_3_/Gln-TSC NPs (Fig. 10).

## Discussion

The frequency of prostate cancer incidence and mortality has been increasing in recent years and thus, finding novel anticancer agents to treat this disease is the goal of many research projects^1^. Nanotechnology products have received great attentions to be used in medicine, especially in cancer treatment. Due to the large surface area, nano-scale compounds could efficiently distribute in the host organs, reach their target sites and exert their cytotoxic effects on the target cells. However, the toxic side-effects of such compounds on the host’s organs are the major barrier to their development and application of them in cancer chemotherapy. In this regard, the preparation of complex compounds containing different effective molecules could increase the efficiency and reduce undesirable features of nanotechnology products. In the current work, we aimed to prepare bismuth oxide NPs, functionalize them with glutamine, and conjugate it to TSC. Then, the effect of the Bi_2_O_3_/Gln-TSC NPs on the viability and apoptosis pathway of prostate cancer cells was evaluated.

Identity of the fabricated Bi_2_O_3_/Gln-TSC NPs were confirmed using the physiochemical assays. The FT-IR and XRD assays confirmed the proper functionalization and synthesis of the NPs. The zeta potential and XRD revealed the stability and low aggregation of the NPs. Further, the nano-scale size and purity of the NPs were confirmed by the electron microscopy and EDX-mapping analyses.

The MTT assay revealed that the fabricated Bi_2_O_3_/Gln-TSC NPs were considerably more cytotoxic for the prostate cancer cells than normal human cells. Also, Bi_2_O_3_/Gln-TSC NPs were more cytotoxic on cancer cell line than Bi_2_O_3_/Gln NPs. Two main hypotheses could be introduced for the higher susceptibility of prostate cancer cells: (1) high proliferation rate and nutrient intake by cancer cells; (2) higher permeability of cancer cells. Due to the high proliferation rate, cancer cells have considerably higher membrane permeability than normal cells to accelerate cell nutrient intake^26^. However, the increased cell permeability could facilitate the penetration of anticancer drugs into the cell. Moreover, glutamine, as an essential amino acid, is considered an essential nutrient for human cells. Due to the higher metabolic rate, cancer cells seem to have a higher demand for such nutrients^27^. The amino acid glutamine plays a key role in the metabolism of highly proliferating cells. During malignant transformation, cancer cells modify the consumption and processing of glutamine to sustain cell growth and proliferation. In some cases, these cancer cells become addicted to glutamine^28^. Thus, targeting the metabolism of glutamine has been developed during last years as a potential strategy against cancer. Therefore, the higher susceptibility of prostate cancer cells than normal cells could be associated with the increased uptake of the Bi_2_O_3_/Gln-TSC NPs into the cancer cells. Previous studies reported that exposure to bismuth NPs could generate radical oxygen species. The generated oxidative stress could damage major cell components, including cell membrane, nucleic acids, enzymes, etc. In addition, previous studies reported that TSC and its derivatives could interrupt the proliferation of eukaryote cells via the inhibition of the enzymes associated with nucleic acid synthesis and replication^14,29,30^.

Evaluation of the frequency of apoptosis in the NP treated and control cells showed that Bi_2_O_3_/Gln-TSC considerably increased the frequency of cell apoptosis. The increase of cell apoptosis suggests that Bi_2_O_3_/Gln-TSC could arrest the cell cycle and initiate cell apoptosis. To investigate this hypothesis, the expression of the apoptotic genes and activity of Caspase-3 enzyme in NPs treated cells were investigated. Real-time PCR assay showed that the expression of *CASP8*, *Bcl-2,* and *BAX* genes in NPs treated cells were significantly increased, while exposure to the NPs downregulated the gene. Caspase-8 is an effector molecule in the extrinsic apoptotic signaling pathway. The generation of oxidative stress in the extracellular environment of NP s treated cells could result in the activation of Caspase-8 protein, which triggers the activation of apoptosis pathways via death receptors^31,32^. Therefore, the increased expression of *CASP8* gene suggests the upregulation of the extrinsic apoptotic pathway that may be associated with the cytotoxic effects of the NPs. Also, the activity of Caspase-3 enzyme as the activator of the caspase-dependent apoptotic pathway was significantly increased upon exposure to Bi_2_O_3_/Gln-TSC NPs^33^. The increased activity of Caspase-3 enzyme reinforces the role of Caspases in the initiation of the apoptotic pathways in the NPs treated cells.

The expression of the *BAX* gene in NPs treated cells was increased significantly while exposure to Bi_2_O_3_/Gln-TSC slightly increased the expression of *Bcl-2*. The BAX protein is regarded as a fundamental effector in the intrinsic apoptosis signaling pathways^34^. In contrast, the BCL2 protein mainly acts as the inhibitor of BAX molecules. The considerable increase of the *BAX*/*Bcl-2* ratio could suggest the activation of both intrinsic and extrinsic pathways of apoptosis in NPs treated cells. In accordance with the present results, previous studies have demonstrated that the *BAX*/*Bcl-2* ratio > 1 can induce the apoptosis^35,36^.

The Hoechst staining of the cells after treatment with Bi_2_O_3_/Gln-TSC NPs indicated the apoptosis features in the exposed cells. In agreement with the flow cytometry and molecular assays, the Hoechst staining confirmed the role of apoptosis in the death of prostate cancer cells in NPs exposed cells.

This work showed that Bi_2_O_3_/Gln-TSC was highly effective against prostate cancer cells. The cytotoxic potential of Bi_2_O_3_/Gln-TSC could be associated with synergism of all components, including Bi_2_O_3_, glutamine, and TSC. As described above, cancer cells have high proliferation rate and nutrient uptake^26^. Since glutamine is an essential nutrient for human cells, it may facilitate internalization of the Bi_2_O_3_/Gln-TSC into the cancer cells^27^. Inside the cells, other components, including Bi_2_O_3_ and TSC could exert their cytotoxic effects by generation of oxidative stress and inhibition of cell enzymes, respectively. Therefore, the synergistic effect of Bi2O3/Gln-TSC components could induce apoptotic signaling pathways in prostate cancer cells.

## Conclusion

In this study, Bi_2_O_3_/Gln-TSC NPs were fabricated and its anticancer effect on prostate cancer cells was investigated. Our results revealed that the synthesized NPs were considerably more cytotoxic for cancer cells than normal human cells and the activation of cell apoptosis through both extrinsic and intrinsic pathways was suggested as the main cytotoxic mechanism of the NPs. Further investigations in in-vivo models could elucidate the potential of Bi_2_O_3_/Gln-TSC NPs to be applied in anticancer chemotherapy.

## Supplementary Information

Supplementary Information.

## Data Availability

The datasets generated during the current study are available from the corresponding author on reasonable request.
