# Supplementary figures and images for: Synthesize of Bi2O3/Gln-TSC nanoparticles and evaluation of their toxicity on prostate cancer cells and expression of CASP8, BAX, and Bcl-2 genes
Source: Sci Rep. 2022 Dec 8;12:21245. doi: 10.1038/s41598-022-25360-6 (PMC9731994; doi:10.1038/s41598-022-25360-6)

Primer efficiency *CASP 8* gene:
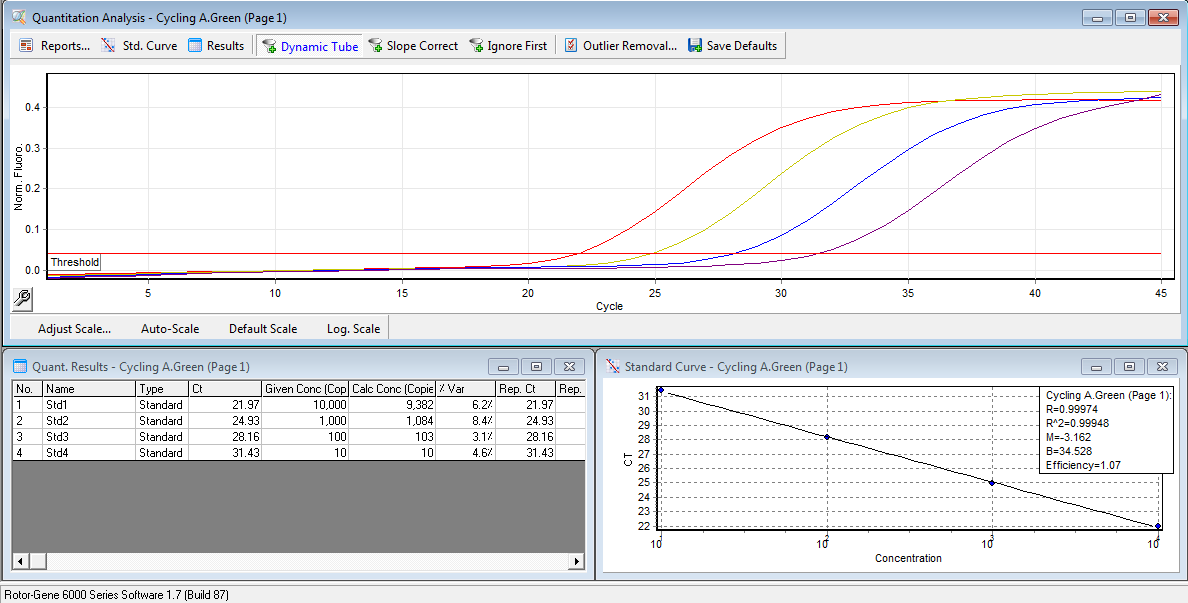


Primer efficiency *BAX* gene:


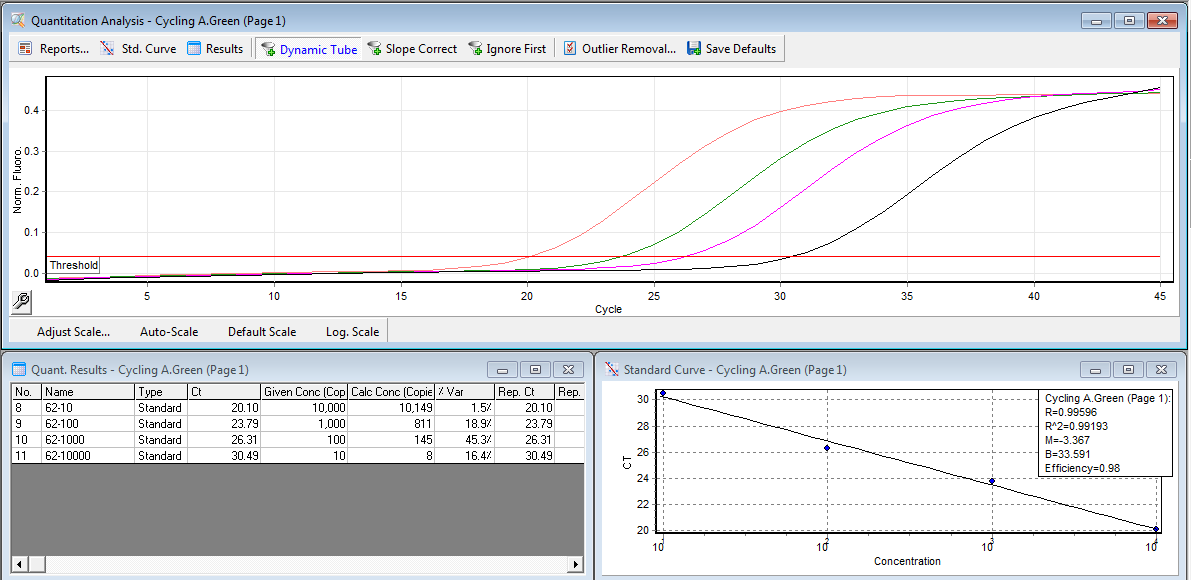


Primer efficiency *BCL2* gene:
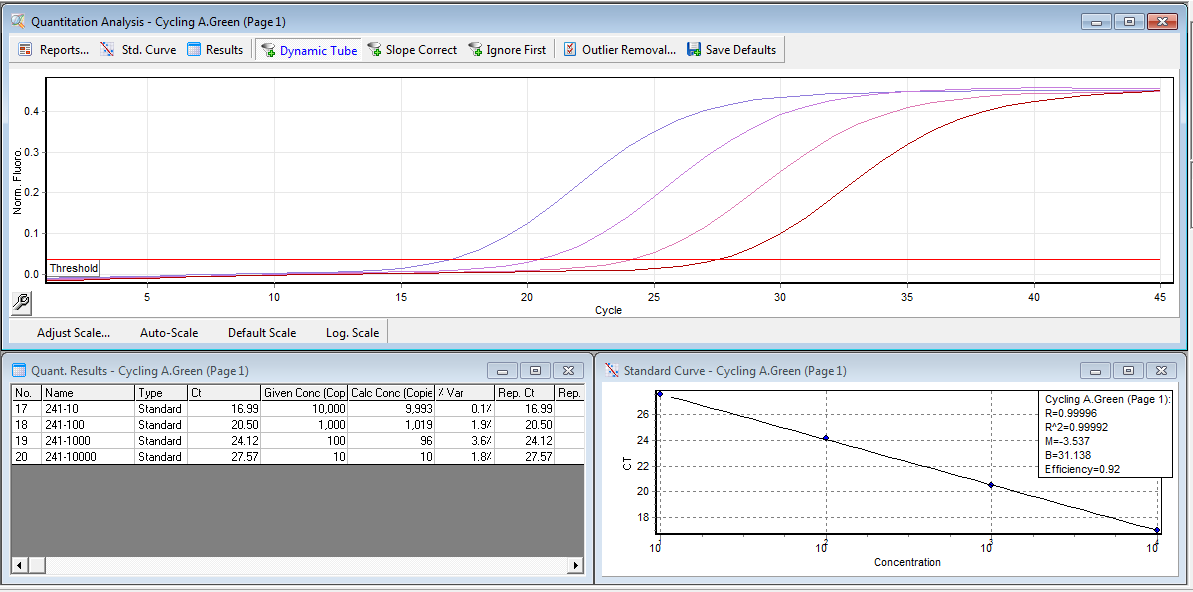

Supplement: Supplementary file 1 — Supplementary Information. [file 41598_2022_25360_MOESM1_ESM.docx]
